# Nano-Delivery System for the Prevention and Control of the Disease

**DOI:** 10.3390/molecules31091448

**Published:** 2026-04-27

**Authors:** Jianxin Zhou, Yalan Mo, Mingfeng Feng, Wenchang Zhang, Chaonan Li, Zhuqing Li, Binghong Jia, Xiaogang Li, Yiping Liu

**Affiliations:** 1 Hunan Provincial Engineering Research Center for Agricultural Pest Early Warning and Control, College of Plant Protection, Hunan Agricultural University, Changsha 410000, China; zhoujianxin@stu.hunau.edu.cn (J.Z.); myl12@stu.hunau.edu.cn (Y.M.);; 2 Agricultural Technology Extension Center of Jianghua Yao Autonomous County, Yongzhou 425500, China; 3 People’s Government of Jinhe Town, Mayang Miao Autonomous County, Huaihua 419402, China; 4 College of Chemistry and Chemical Engineering, Central South University, Changsha 410000, China; 5 Institute of Cotton and Sericulture, Hunan Academy of Agricultural Sciences, Changsha 410127, China

**Keywords:** kiwifruit, soft rot disease, nanoliposomes, carvacrol, nano-delivery

## Abstract

Kiwifruit soft rot is a major cause of postharvest loss owing to rapid fruit decay during storage. This study focused on kiwifruit soft rot during the postharvest storage stage, when fungal development may be promoted by room temperature and high humidity. Soft rot symptoms were observed in the pericarp and fruit flesh. In this study, carvacrol-loaded nanoliposomes (CAR@NL) were prepared by an O/W emulsification–solvent evaporation method to control kiwifruit soft rot. The physicochemical properties of CAR@NL were characterized by laser particle size analysis, Fourier transform infrared spectroscopy (FTIR), and transmission electron microscopy (TEM). Their antifungal activity and preservation efficacy were evaluated by in vitro antifungal assays and fruit storage experiments. The prepared CAR@NL showed an average particle size of approximately 280 nm, an encapsulation efficiency of 85.75%, and a drug loading capacity of 20.14%, along with favorable sustained-release properties. CAR@NL exhibited strong antifungal activity, with an EC_50_ value of 41.76 mg/L. DAPI staining indicated no obvious effect on fungal DNA, whereas propidium iodide (PI) staining revealed increased fluorescence intensity with increasing concentration and treatment time, indicating disruption of hyphal membrane integrity and severe structural damage. Flow cytometric analysis further showed that, at 50 mg/L, the total apoptosis rate was 2.96% in the untreated control group, 5.22% in the CAR@NL-treated group, and 33.6% in the carbendazim-treated group, demonstrating the lower cytotoxicity of CAR@NL toward mammalian cells. In addition, CAR@NL showed good stability and preservation performance during fruit storage. Overall, CAR@NL may serve as a safe and effective postharvest agent for the control of kiwifruit soft rot.

## 1. Introduction

Kiwifruit is rich in nutrients and boasts a pleasantly sweet-tart flavor, making it highly favored by consumers [1]. China, as the country with the most diverse kiwifruit varieties and the widest range of cultivated cultivars, holds a unique advantage in this industry [2,3]. However, kiwifruit soft rot has become a major bottleneck constraining the development of China’s kiwifruit industry [4]—causing significant loss rates during fruit transportation and storage, resulting in severe economic damage to the sector [5]. The primary pathogen responsible for this disease is *Botryosphaeria dothidea* [6]. The pathogen lies dormant within the kiwifruit during its early growth stages, invading the fruit only after wounds occur during harvesting, transportation, or storage. Symptoms typically become apparent only when the fruit reaches maturity [7].

Soft rot is the most prevalent disease affecting kiwifruit storage worldwide. With the continuous development of China’s kiwifruit industry, kiwifruit soft rot has gradually attracted increased attention from researchers. Currently, integrated pest management (IPM) serves as the primary approach for controlling kiwifruit soft rot, as no specific pesticides exist for its effective prevention and control [8]. Reports indicate that glycerol can enhance kiwifruit resistance to soft rot [9], while natamycin demonstrates effective control against the disease [10]. However, the practical efficacy of these control methods in real-world applications remains to be developed and validated.

Liposomes are composed of phospholipids and cholesterol as wall materials [11], prepared through methods such as ethanol injection [12], thin-film [13], and solvent diffusion techniques [14]. Liposomes find extensive applications in the biomedical field, playing a particularly crucial role in cancer treatment [15]. Although extensive research has been conducted on liposomes in the medical field, exploration in agriculture remains relatively scarce. However, their unique advantages stemming from nanoscale dimensions—such as size effects and surface effects [16]—are gradually attracting widespread attention from researchers. Liposomes can efficiently penetrate cellular barriers to reach diseased tissues, enabling precise delivery of active ingredients to target sites [17]. This not only significantly enhances the bioavailability of active components but also markedly improves disease control efficacy. The outstanding delivery efficiency of liposomes stems from three core advantages: First, their strong permeation ability due to nanoscale dimensions [18]; second, the high compatibility between their phospholipid bilayer structure and biological membranes [19]; and third, their superior loading capacity for hydrophobic active ingredients [20]. The synergistic effect of these properties makes nanoliposomes a highly promising nanodelivery vehicle in the field of plant protection.

Essential oils (EOs) are a class of highly volatile natural aromatic compounds [21]. Extensive research has confirmed that numerous essential oils exhibit significant inhibitory activity against pathogenic microorganisms such as bacteria and fungi [22]. Their inherent high volatility results in minimal disturbance to plants and the ecological environment during field applications, with no apparent risk of phytotoxicity, demonstrating excellent environmental compatibility. However, the inherent high volatility of EOs results in a short residual efficacy period in actual field control scenarios, presenting a technical challenge that limits their agricultural application and promotion. To address this issue, microencapsulation technology can be employed to encapsulate EOs, thereby reducing their volatility, minimizing losses, and extending their residual efficacy in the field [23]. In contrast, the lipophilic structure of the nano-liposomes developed in this study provides effective protection for the encapsulated EOs’ active components. With their nanoscale particle size, these liposomes enable efficient delivery of key active ingredients to target sites, significantly enhancing pest control efficacy.

This study screened carvacrol (CAR) through plate inhibition assays for its high antibacterial activity against the soft rot pathogen. However, CAR exhibits high volatility and is prone to degradation in complex field environments. To address these limitations, this study developed a carvacrol nanoliposome delivery system, preparing carvacrol nanoliposomes (CAR@NL) via solvent evaporation. The key physicochemical and functional parameters of this nanodelivery system were characterized, including morphology, particle size distribution, encapsulation efficiency, drug loading capacity, and in vitro sustained-release performance. Concurrently, its efficacy against kiwifruit soft rot disease was evaluated. This study aims to develop an efficient and stable plant-derived antimicrobial nanoparticle, thereby providing new insights, strategies, and technical support for the green control of post-harvest diseases in kiwifruit.

## 2. Results and Discussion

### 2.1. Morphological Evaluation

**Figure 1:**
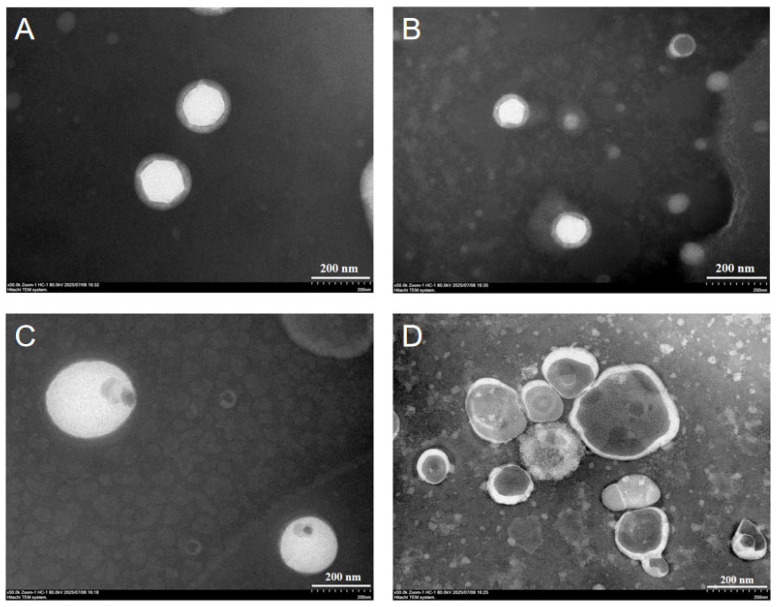
(**A**,**B**): Transmission electron microscopy images of CAR@NL; (**C**,**D**): Transmission electron microscopy images of NL.

Transmission electron microscopy (TEM) characterization revealed significant agglomeration of the nanoliposomes when observed using a Hitachi HT-7800 TEM (Hitachi, Ltd., Tokyo, Japan). In TEM images, the shadowed regions may correspond to collapsed areas of the nanoparticle. In contrast, drug-loaded nanoliposomes (CAR@NL) appear as smooth spherical nanoparticles with no observable aggregation or adhesion (Figure 1A,B). Compared to unloaded nanoliposomes (NL) (Figure 1C,D), drug-loaded nanoliposomes not only exhibit thicker particle wall structures but also demonstrate significantly improved dispersibility. This may be attributed to CAR, as a naturally lipophilic substance, forming a stable encapsulation layer within the bilayer lipid membrane of the liposome. This increases the membrane thickness, thereby enhancing nanoparticle stability, reducing the tendency for particle aggregation, and improving dispersibility.

Yu et al. prepared dual-responsive AVM microcapsules by depositing chitosan and sodium lignosulfonate onto AVM crystals through a layer-by-layer assembly strategy. Compared with their microcapsules, the nanoparticles prepared in this study exhibited a smaller particle size and a more uniform morphology. These characteristics may be favorable for leaf deposition and foliar uptake, and thus may contribute to improved pesticide utilization efficiency [24].

### 2.2. Particle Size

**Figure 2:**
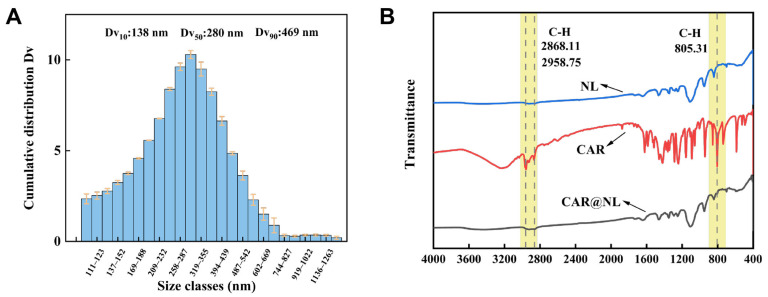
Infrared Spectroscopy and Particle Size Analysis: (**A**): CAR@NL particle size distribution diagram; (**B**): CAR@NL infrared spectrum (CAR@NL: carvacrol nanoliposomes; CAR: carvacrol; NL: unloaded nanoliposomes), *X*-axis: Wavenumber (cm^−1^).

The particle size distribution of CAR@NL was characterized using BT-9300s laser particle size analyzer (Dandong Baite Instrument Co., Ltd., Dandong, China), and all measurements were performed in triplicate (Figure 2A). The volume-based cumulative distribution exhibits a narrow, unimodal profile with a median diameter (Dv_50_) of 280 nm. The particle size distribution spans from 138 nm (Dv_10_) to 469 nm (Dv_90_), and the low polydispersity index of 1.18 indicates a relatively uniform particle size distribution.

Cen et al. developed a photothermal controlled-release microcapsule delivery system for avermectin using chitosan and polydopamine (PDA) as shell materials, and the reported Dv50 of the AVM@CS/CMA/PDA microcapsules was 2.85 µm [25]. In comparison, the nanoparticles prepared in this study showed a more distinct nanoscale feature.

### 2.3. Fourier Transform Infrared (FTIR) 

The interaction between CAR and NL was investigated using Fourier Transform Infrared Spectroscopy (FTIR) (Thermo Fisher Scientific, Waltham, MA, USA) (Figure 2B). Compared to pure CAR, the FTIR spectrum of CAR@NL exhibited significant changes. Specifically, the CAR@NL spectrum completely lacks the characteristic absorption bands corresponding to the asymmetric stretching vibration of the methyl (–CH_3_) group (2868.11 cm^−1^) and the symmetric stretching vibration of the methylene (–CH_2_) group (2958.75 cm^−1^). This phenomenon indicates that the active ingredient molecules have been effectively encapsulated within the nanocarbon black, leading to a significant reduction in the surface concentration of these functional groups. However, a prominent absorption peak at 805 cm^−1^ attributable to the out-of-plane bending vibration of aromatic ring C–H bonds remains detectable. The retention of this specific peak confirms the successful embedding of the active ingredient into the nanoparticle, thereby validating the formation of the CAR@NL composite.

### 2.4. Encapsulation Efficiency (EE) and Drug Loading (DL)

We weighed 0.1 g of CAR@NL and placed it in a beaker. We added 20 mL of methanol solution and sonicated for 10 min. We prepared three parallel samples using the same method. The resulting samples were analyzed by Waters e2695 high-performance liquid chromatograph (WATERS Corporation, Milford, MA, USA). The encapsulation efficiency (EE) and drug loading (DL) of CAR@NL were calculated using Equations (1) and (2), respectively. The results indicated an encapsulation efficiency of 85.75% and a drug loading of 20.14%. Compared with the microcapsules reported by Cui et al. (encapsulation efficiency, 64.31%) [26], the nanoparticles prepared in this study exhibited a higher encapsulation efficiency of 85.75%, indicating superior loading capacity for the active ingredient. This result suggests that the present nanosystem holds promise for practical application, although its overall application potential should be further evaluated in combination with stability, release behavior, and bioactivity.

### 2.5. Release Characteristics

The results of the CAR@NL drug sustained-release experiment conducted in a 30% methanol solution demonstrate that this nanoparticle exhibits distinct sustained-release characteristics. During the initial phase, drug release occurs at a relatively slow rate. Over time, the release rate gradually increases until reaching a plateau phase after approximately 50 h, revealing a relatively stable release trend. The overall release process conformed to a typical sustained-release pattern, with the release rate gradually decreasing over time. These results indicate that the drug release behavior of CAR@NL in methanol solution is controllable, achieving a cumulative release rate of 77% after 80 h. This demonstrates that CAR@NL possesses excellent sustained-release properties.

**Figure 3:**
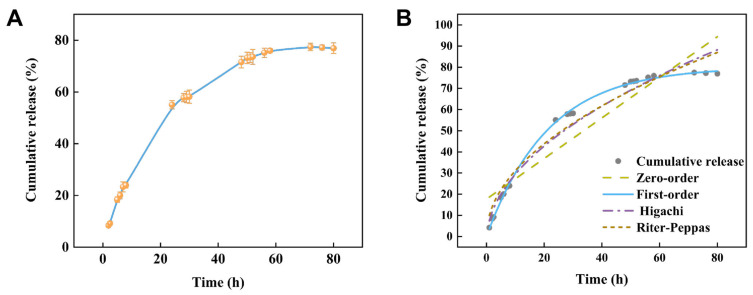
(**A**) Sustained-release profile of CAR@NL, with data presented as mean ± SD (*n* = 3); (**B**) Kinetic model fitting of CAR@NL.

As shown in Figure 3 and Table 1, the release behavior of CAR@NL was best described by the first-order kinetic model, which showed the highest coefficient of determination (R^2^ = 0.99855). By comparison, the Ritger–Peppas and Higuchi models showed lower goodness of fit, with R^2^ values of 0.96241 and 0.96442, respectively, whereas the zero-order model exhibited the poorest fit (R^2^ = 0.86639). These results indicate that the release of CAR@NL follows a first-order release mechanism, suggesting that the release rate is dependent on the concentration of the remaining active compound in the nanoparticle system.

Furthermore, according to the Ritger–Peppas model, the release exponent n for CAR@NL was 0.49. Since this value is lower than 0.5, the release process can be characterized as Fickian diffusion [27]. Therefore, kinetic model fitting not only identifies the most appropriate mathematical model for describing the release behavior of CAR@NL, but also provides mechanistic insight into its diffusion-controlled release characteristics, which is important for understanding its potential application as a controlled-release nanoparticle system.

### 2.6. Storage Stability

**Figure 4:**
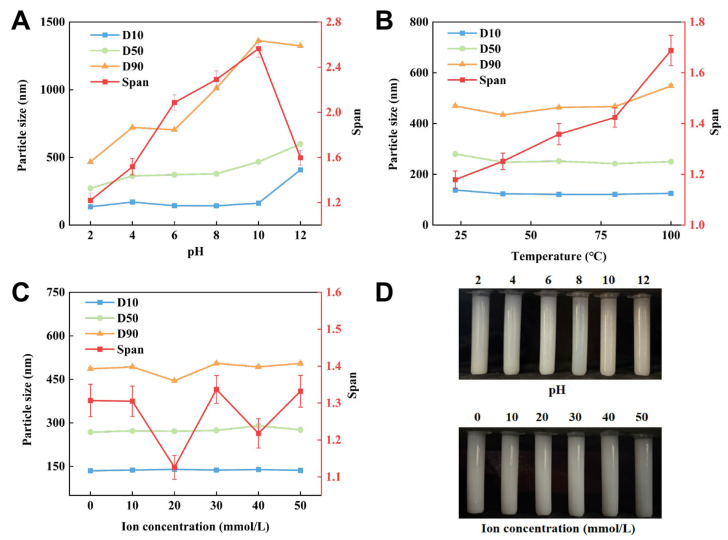
Stability testing with data presented as mean ± SD (*n* = 3): (**A**): pH stability; (**B**): Temperature stability; (**C**): Ion stability; (**D**): pH and ion stability CAR@NL state.

Systematic evaluation of the ionic, pH, and thermal stability of CAR@NL showed that the nanoparticle remained stable over a sodium chloride ion concentration range of 0–50 mmol/L (Figure 4). Specifically, D10 increased slightly from 135 to 136 nm (0.74%), D50 from 268 to 276 nm (2.98%), and D90 from 486 to 505 nm (3.91%), indicating negligible changes in particle size distribution under different ionic strengths.

CAR@NL also exhibited good thermal stability over the temperature range of 23–100 °C. D10 decreased from 138 to 125 nm (−9.42%), and D50 decreased from 280 to 250 nm (−10.71%), while D90 increased from 469 to 548 nm (16.84%). Overall, no marked variation in particle size was observed, suggesting that the nanoparticle maintained relative stability under thermal stress.

In contrast, the stability of CAR@NL was strongly affected by pH. As the pH increased, the particle size increased markedly, with D10 rising from 135 to 408 nm (202.22%), D50 from 272 to 599 nm (120.22%), and D90 from 467 to 1324 nm (183.51%). Meanwhile, the nanoparticle color changed from white to dark gray, suggesting physicochemical instability under alkaline conditions. CAR@NL was substantially more stable under acidic conditions. These results suggest that CAR@NL should not be mixed with alkaline pesticides in practical applications.

The pH-dependent particle growth may be attributed to the deprotonation of carboxyl groups in the fatty acid-based lipid molecules constituting CAR@NL under alkaline conditions. This deprotonation likely increases bilayer permeability and disrupts membrane packing, leading to a transition from a compact and ordered structure to a looser and more disordered state, which is reflected by the increase in particle size [28,29].

### 2.7. Antimicrobial Properties

**Figure 5:**
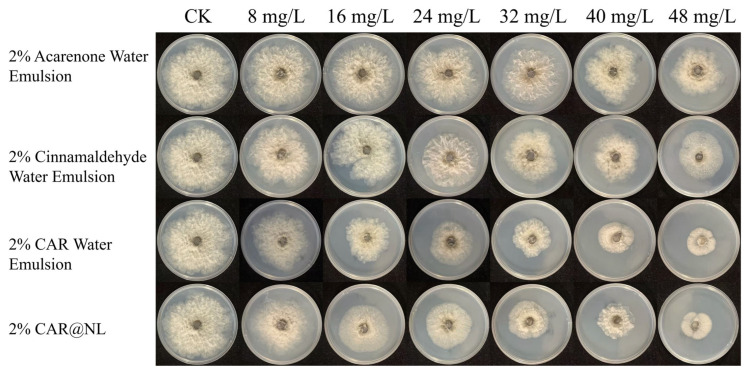
Plate antimicrobial assay showing the activity of 2% Acarenone Water Emulsion, 2% Cinnamaldehyde Water Emulsion, 2% CAR Water Emulsion, and 2% CAR@NL.

The mycelial growth rate method was employed to determine the potency of 2% Acarenone water emulsion, 2% cinnamaldehyde water emulsion, 2% CAR water emulsion, and 2% CAR@NL against the pathogen causing kiwifruit soft rot (Figure 5). Results are shown in Table 2. The EC_50_ of 2% Acarenone water emulsion was 134.508 mg/L, while the EC_50_ values for the 2% cinnamaldehyde water emulsion, 2% CAR water emulsion, and 2% CAR@NL were 92.664 mg/L, 34.904 mg/L, and 41.761 mg/L, respectively.

Comprehensive comparison of the toxicity results across different formulations revealed that the CAR water emulsion exhibited stronger antibacterial activity. Therefore, it was selected as the active ingredient for encapsulation within nanoliposomes. Results demonstrated that CAR@NL possesses good biological activity against the pathogen causing kiwifruit soft rot disease, indicating its potential as a novel nanoparticle for the control of kiwifruit soft rot disease.

CAR water emulsion exhibited stronger antibacterial activity than CAR@NL. This difference may be attributed to the encapsulation of CAR within the nanoparticle system. The antibacterial effect of CAR largely depends on its rapid interaction with the bacterial cell membrane, leading to membrane depolarization, membrane disruption, and leakage of intracellular contents [30]. Free CAR can directly contact bacterial cells upon addition, whereas encapsulated CAR must first be released from the nanoparticles, which may reduce the immediate availability of the active compound and consequently weaken its antibacterial activity.

### 2.8. Effectiveness of CAR@NL on Fruit Preservation

**Figure 6:**
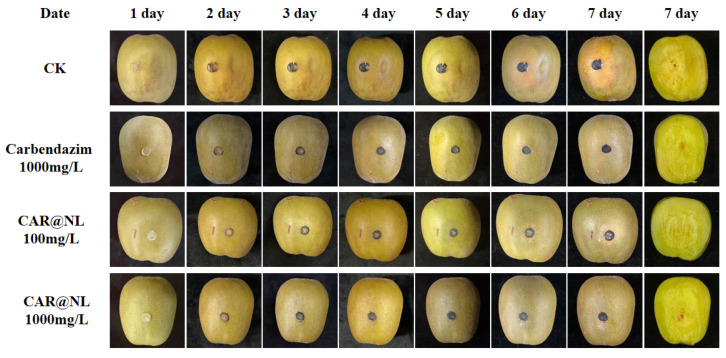
Kiwifruits treated with different concentrations of carbendazim and CAR@NL.

Similar analyses have also been reported in other fruits. For example, Shen et al. described comparable postharvest fungal infection patterns in tangerine [31], while Pu et al. reported similar disease progression in pear [32]. After treating kiwifruit and observing disease progression, water-soaked lesions began appearing in the CK group on day 3 and gradually expanded with prolonged incubation (Figure 6). In contrast, distinct lesions emerged in the 100 mg/L CAR@NL, 1000 mg/L CAR@NL, and 1000 mg/L carbendazim groups on days 4, 6, and 7, respectively. Upon peeling the kiwifruit skin for examination, the CK group exhibited significantly larger lesion areas than the 100 mg/L CAR@NL, 1000 mg/L CAR@NL, and 1000 mg/L carbendazim-treated groups by day 7. Lesion sizes were similar between the 1000 mg/L CAR@NL and 1000 mg/L carbendazim treatments. Collectively, these results indicate that both 1000 mg/L CAR@NL and 1000 mg/L carbendazim effectively inhibit Botryosphaeria dothideniae infection, significantly reduce kiwifruit disease incidence, slow lesion expansion rates, and demonstrate good inhibitory effects against kiwifruit soft rot.

### 2.9. Cell Membrane Permeability and Nuclear Staining

**Figure 7:**
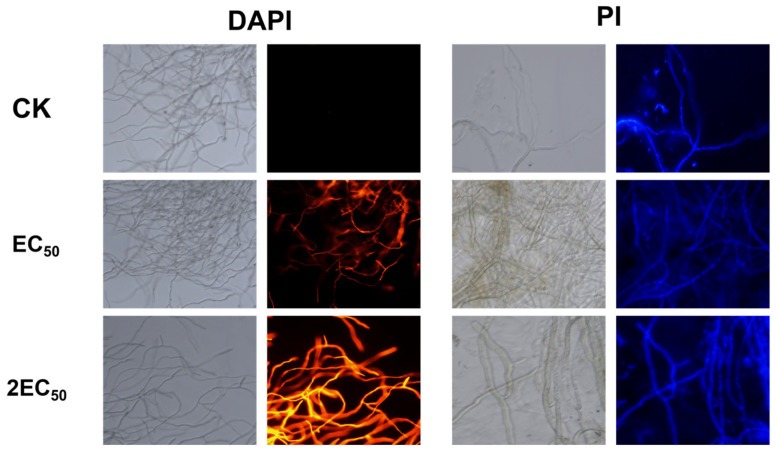
Effects of CAR@NL on hyphal membrane integrity and nuclear morphology of *Botryosphaeria dothidea* as revealed by PI and DAPI staining.

DAPI staining revealed no obvious change in the DNA-associated fluorescence of *Botryosphaeria dothidea* hyphae after CAR@NL treatment, indicating that CAR@NL had no significant effect on hyphal DNA. In contrast, PI staining clearly demonstrated membrane damage in *B. dothidea* hyphae following treatment with CAR@NL. The PI fluorescence intensity increased in a concentration- and time-dependent manner, and the strongest fluorescence signal was observed at 2EC_50_, whereas almost no fluorescence was detected in the control. Because PI selectively enters cells with damaged membranes, these results indicate that CAR@NL rapidly compromised hyphal membrane integrity. Collectively, the DAPI and PI staining results (Figure 7) suggest that the antifungal activity of CAR@NL is mainly associated with disruption of hyphal structural integrity, leading to severe hyphal damage.

### 2.10. Biological Safety

**Figure 8:**
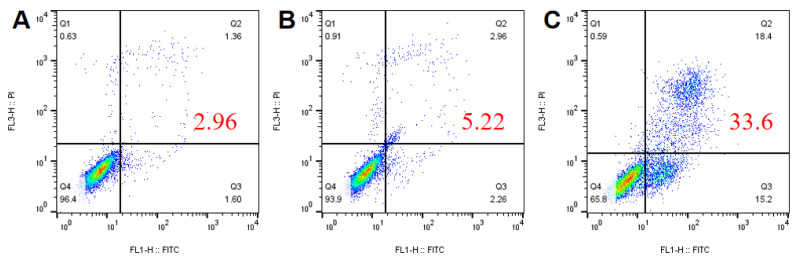
Effects of CAR@NL, carbendazim (**A**) Untreated control group, (**B**) CAR@NL group, (**C**) carbendazim group.

Considering the potential adverse effects of pesticide exposure on human health, the apoptotic response induced by CAR@NL was further investigated (Figure 8). Flow cytometry results revealed that, at 50 mg/L, the total apoptosis rate was 2.96% in the untreated control group and 5.22% in the CAR@NL-treated group, whereas cells exposed to carbendazim exhibited a significantly elevated apoptosis rate of 33.6%. These results suggest that CAR@NL preserves effective antifungal activity while causing minimal apoptosis in mammalian cells, underscoring its promise as a safer alternative to conventional fungicides. Representative apoptosis-related response values reported for mammalian cells in the reference by Feng et al. were included as a reference to interpret the present result [33]. Compared with these reported values, our observed value indicates CAR@NL with a relatively low apoptosis-related risk.

## 3. Materials and Methods

### 3.1. Materials

SK-5945 (primarily polyoxyethylene sorbitan monooleate (Tween 80–type), Non-ionic surface active agent) was purchased from Shanghai Xingfei Chemical Co., Ltd. (Shanghai, China); AEO-3P (primarily lauryl ether phosphate, Anionic surface active agent) was purchased from Jiangsu Hai’an Petrochemical Factory (Haian City, China); Dichloromethane (AR), Carvacrol (purity ≥ 98%), soybean lecithin (Biotech, 758.06 MW), Methanol (HPLC) and cholesterol (purity ≥ 99%, 386.65 MW) were purchased from Shanghai McLean Biochemical Technology Co., Ltd. (Shanghai, China); Potassium bromide (SP, purity ≥ 99.5% 119 MW) was purchased from Shanghai Aladdin Reagent Co., Ltd. (Shanghai, China); Carbendazim was purchased from Sichuan Runer Technology Co., Ltd. (Chengdu, China) (low toxicity). Other resources included the following: Waters e2695 high-performance liquid chromatograph (WATERS Corporation, Milford, MA, USA); Hitachi HT-7800 transmission electron microscope (Hitachi, Ltd., Tokyo, Japan); SCIENTZ-10N/C freeze dryer (Ningbo Xinzhi Biotechnology Co., Ltd., Ningbo, China); Magnetic stirrer (Henan Aibote Technology Development Co., Ltd., Zhengzhou, China); 5804R high-speed bench-top large-capacity centrifuge (Eppendorf AG, Hamburg, Germany); BT-9300s laser particle size analyzer (Dandong Baite Instrument Co., Ltd., Dandong, China); and Fourier Transform Infrared Spectrometer (Thermo Fisher Scientific, Waltham, MA, USA).

### 3.2. Preparation of CAR@NL

CAR@NL refers to carvacrol-loaded nano-liposomes (liposome-like lipid nanoparticle) prepared via an O/W emulsification–solvent evaporation method, with soybean lecithin and cholesterol as the lipid matrix and CAR incorporated into the lipid/organic phase.

The O/W emulsification–solvent evaporation method employed in this study differs fundamentally in principle from the conventional ethanol injection method [34]. In the latter, an ethanolic solution of lipids and drugs is injected into an aqueous phase under stirring, where liposomes form spontaneously due to the rapid dilution of ethanol; residual solvent is subsequently removed via rotary evaporation or dialysis.

In contrast, referring to the nanoparticle preparation protocol of Marta Szczęch et al., this study selected dichloromethane [35], which is highly volatile at room temperature, as the organic solvent. The core principle relies on forming an emulsion between the immiscible organic (oil) and aqueous phases via mechanical shearing. The subsequent evaporation of the organic solvent induces the directional alignment and self-assembly of lipid molecules at the interface, resulting in the formation of vesicular structures. Furthermore, based on the report by C. Has et al. that surfactants facilitate the reduction of vesicle size [36], a binary surfactant system comprising SK-5945 and AEO-3P was utilized in this study.

**Figure 9:**
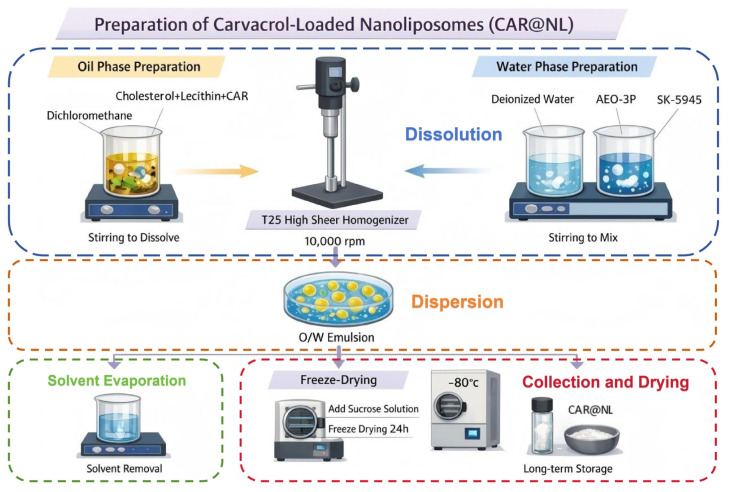
CAR@NL preparation flowchart.

Following the method of Yousefi et al. for preparing liposomes with certain modifications, the O/W emulsification–solvent evaporation method was employed to synthesize CAR@NL [37]: Weigh 2 g cholesterol and 5 g soybean lecithin, dissolve in dichloromethane, and stir with a magnetic stirrer until cholesterol and soybean lecithin are completely dissolved in 20 mL dichloromethane. Then, add 2 g CAR to ensure its complete dissolution in the oil phase, yielding an CAR-containing oil phase for later use. Weigh 87 g of deionized water, add 2 g of AEO-3P and 2 g of SK-5945, and stir with a magnetic stirrer to obtain the aqueous phase for later use. Use a T25-digital high-speed shear mixer to rapidly shear the aqueous phase at 10,000 rpm. While the aqueous phase is maintained under high-shear homogenization (10,000 rpm), the oil phase is added dropwise over 20 min. After adding the oil phase, rapidly shear for 20 min to obtain an O/W emulsion. Stir the final sheared solution on a magnetic stirrer until all dichloromethane has completely evaporated, yielding CAR@NL. Take 20 mL of CAR@NL, add 10 mL of 1% sucrose solution as an antifreeze agent, then freeze-dry for 24 h using a freeze dryer to remove excess moisture. Store at −80 °C for future use. This preparation process is shown in Figure 9.

### 3.3. Morphological Evaluation of CAR@NL

To observe the morphological characteristics of CAR@NL, the appearance and morphology of the microspheres were examined using a Hitachi HT-7800 transmission electron microscope (TEM), and images were acquired. One sample each of CAR@NL and NL was prepared for TEM observation using a negative-staining method [38]. Then, 10 µL of each sample was deposited onto a copper grid and allowed to stand at room temperature for 1 min to facilitate adsorption. Excess liquid was removed from the edge with filter paper, followed by the addition of 10 µL of phosphotungstic acid staining solution for negative staining. After incubation at room temperature for 1 min, the excess stain was wicked away with filter paper, and the grid was air-dried at room temperature for several minutes. After complete drying, the samples were observed and imaged under TEM at an accelerating voltage of 80–120 kV, and the acquired images were used for subsequent analysis.

### 3.4. Particle Size of CAR@NL

Particle size distribution is a critical factor determining liposome stability [39]. To measure the particle size of CAR@NL, freshly prepared CAR@NL was diluted to an appropriate concentration with deionized water prior to testing using a laser particle size analyzer.

### 3.5. Fourier Transform Infrared (FTIR) Analysis

To demonstrate the loading of CAR into NL, Fourier Transform Infrared Spectroscopy was employed for absorbance analysis, referencing Eusepi et al. with modifications [40]. CAR@NL and NL were analyzed via the potassium bromide (KBr) pellet method. A 2 mg sample was mixed with 200 mg KBr in an agate mortar and thoroughly ground to obtain a fine, uniform powder. Subsequently, the powder mixture was subjected to pressure to form thin, transparent pellets. The absorbance of each sample was then measured using a Fourier Transform Infrared Spectrometer.

### 3.6. EE and DL

Refer to the method of Alarjah et al. and make appropriate modifications [41]. A standard curve for CAR is constructed using a Waters e2695 high-performance liquid chromatography (HPLC) system. Accurately weigh 100 mg of CAR reference standard, dissolve in methanol, mix thoroughly, and dilute to a 100 mL volumetric flask to prepare a stock solution with a mass concentration of 1000 mg/L. Accurately pipette appropriate volumes of the stock solution and sequentially dilute with methanol to prepare a series of standard solutions at 5, 10, 20, 40, 60, 80, and 100 mg/L. Transfer each standard solution to an HPLC injection vial and analyze under identical chromatographic conditions. Plot peak area versus mass concentration to establish the CAR standard curve. Chromatographic conditions were as follows: Waters TC-C18 stainless steel column (4.6 mm × 250 mm, 5 µm); mobile phase methanol:water (*v*/*v* = 75:25); flow rate 1.0 mL/min; detection wavelength 226 nm; injection volume 20 µL; and column temperature 35 °C.

The encapsulation efficiency (EE) and drug loading (DL) of CAR in CAR@NL were indirectly calculated by determining the content of unencapsulated CAR. This method references the report by Maritim et al. and incorporates appropriate modifications [42]. Freshly prepared CAR@NL suspensions were placed in a high-speed bench-top large-capacity centrifuge and centrifuged at 13,000 r/min for 1 h at 4 °C. After centrifugation, the supernatant was collected, filtered through a 0.22 µm Millipore membrane (Billerica, MA, USA), and analyzed by HPLC to determine the unencapsulated CAR content. The EE of CAR@NL was calculated using Equation (1), and the DL was calculated using Equation (2).

(1) $$\mathrm{EE}=\frac{\mathrm{Active}\;\mathrm{ingredient}\;\mathrm{content}\;\mathrm{within}\;\mathrm{the}\;\mathrm{capsule}}{\mathrm{Total}\;\mathrm{Active}\;\mathrm{Ingredient}\;\mathrm{Content}}\times 100\%$$

(2) $$\mathrm{DL}=\frac{\mathrm{Active}\;\mathrm{ingredient}\;\mathrm{content}\;\mathrm{within}\;\mathrm{the}\;\mathrm{capsule}}{\mathrm{Overall}\;\mathrm{Preparation}\;\mathrm{Quality}}\times 100\%$$

### 3.7. Release Characteristics

The sustained-release performance of CAR@NL was evaluated using the dialysis bag method, adapted from the approach described by Walunj et al. with appropriate modifications [43]. Two grams of CAR@NL were weighed and placed into a dialysis bag, which was sealed and immersed in a beaker containing 500 mL of 30% methanol. The bag was placed on a magnetic stirrer and stirred at 200 r/min under ambient temperature conditions. At predetermined intervals, 2 mL of supernatant was sampled (replenishing 2 mL of 30% methanol after each sampling) for liquid chromatography analysis. Each sample was replicated three times, and the average value was recorded.

The CAR@NL release curve was plotted. Common kinetic release models—zero-order, first-order, Higuchi, and Ritger–Peppas—were selected to fit and analyze the CAR@NL release behavior [44].

### 3.8. Storage Stability

#### 3.8.1. Ion Strength Stability

Refer to the method of Wang et al. and make appropriate modifications [45]. Transfer CAR@NL into centrifuge tubes, replicate each sample three times, add sodium chloride solutions of varying ionic concentrations, allow to stand at room temperature for 30 min, observe whether the solution separates into layers, measure the CAR@NL particle size using a laser particle size analyzer, and analyze changes in CAR@NL particle size.

#### 3.8.2. pH Stability

Refer to the method of Ren et al. and make appropriate modifications [46]. Transfer CAR@NL into centrifuge tubes, and replicate each sample three times. Adjust the pH of CAR@NL to different values using sodium hydroxide or hydrochloric acid. Allow the solution to stand at room temperature for 30 min, then observe whether it separates into layers. Measure the particle size of CAR@NL using a laser particle size analyzer and analyze the changes in CAR@NL particle size.

#### 3.8.3. Temperature Stability

Refer to the method of Wang et al. and make appropriate modifications [47]. Transfer CAR@NL into centrifuge tubes and replicate each sample three times. Stir using a magnetic stirrer and heat to different temperatures. After heating in a water bath for 30 min, observe whether the solution separates into layers. Test the CAR@NL particle size using a laser particle size analyzer and analyze changes in CAR@NL particle size.

### 3.9. CAR@NL Flat Plate Bactericidal Test

To determine the inhibitory effect of CAR@NL on the pathogen causing kiwifruit soft rot, an in vitro bioassay was conducted, following the method described by Li et al. with appropriate modifications [48]. Sterile PDA medium was prepared and supplemented with water-emulsifiable formulations of CAR, cinnamaldehyde, and Litsea cubeba oil, along with CAR@NL and NL. to create six concentrations of drug-containing plates: 8 mg/L, 16 mg/L, 24 mg/L, 32 mg/L, 40 mg/L, and 48 mg/L. A control group without any drug was also established. Each treatment was replicated three times and incubated upside down in a 26 °C incubator under dark conditions for 5 days. Colony diameters were measured using the cross-count method, and the average values were calculated. Experimental data was performed using SPSS 27.0.

### 3.10. Application in Fruit Preservation

Refer to the method of Wu et al. and make appropriate modifications [49]. Kiwifruits with a size range of 100–120 mm and similar weight, free from decay, and with comparable firmness were selected as the experimental materials. Hongyang kiwifruits (*Actinidia chinensis*) purchased from a local market were used in this study. Wash the surface impurities with tap water, then disinfect the fruit surface by spraying with a 75% (*v*/*v*) ethanol solution. Rinse three times with sterile water and allow to air dry naturally. Apply the test agent uniformly to the kiwifruit surface via spraying until fully wetted, then allow to air-dry at room temperature. Use a sterile needle to puncture the skin at the equatorial region of each fruit, creating one artificial wound. Subsequently, inoculate each wound with a uniformly sized kiwifruit soft rot pathogen cake. Store the treated kiwifruit at 25 °C for 7 days, documenting fruit rot progression over this period. Use sterile water-treated fruit as the control, with three replicates per treatment.

### 3.11. Cell Membrane Permeability and Nuclear Staining Assays

Cell membrane permeability and nuclear morphology were evaluated using propidium iodide (PI) and 4′,6-diamidino-2-phenylindole (DAPI) staining, respectively. *Botryosphaeria dothidea* was incubated in potato dextrose broth (PDB) at 28 °C and 180 rpm for 24 h. Subsequently, CAR@NL was added to achieve final concentrations corresponding to the EC_50_ and 2EC_50_ values, with deionized water serving as the control. After treatment for 30 min, 0.1 g of mycelia was collected and stained separately with PI or DAPI for 30 min in the dark. The samples were washed three times with 0.1 M phosphate-buffered saline (PBS) and observed under a fluorescence microscope.

### 3.12. Biological Safety Evaluation

Cell apoptosis was analyzed by Annexin V-FITC/PI double staining using flow cytometry. BEAS-2B cells in the logarithmic growth phase were seeded into 6-well plates at a density of 4 × 10^5^ cells per well and cultured overnight at 37 °C under 5% CO_2_ to allow adherence. Untreated cells served as the control, while the treatment groups were exposed to CAR@NL or carbendazim at 50 mg/L for 24 h. After incubation, the cells were harvested, washed twice with ice-cold PBS, and resuspended in 300 µL Binding Buffer. Then, 5 µL Annexin V-FITC was added, and the cells were incubated in the dark on ice for 15 min, followed by the addition of 10 µL PI. The stained cells were analyzed by flow cytometry within 1 h.

### 3.13. Statistical Analysis

All measurements were performed in triplicate, and the resulting data were ex-pressed as mean ± standard deviation. Statistical analyses were performed using SPSS Statistics 27.0 with one-way analysis of variance. Significance levels were evaluated using Duncan’s new complex polarity method (*p* < 0.05).

## 4. Conclusions

This study used an O/W emulsification–solvent evaporation method to prepare CAR@NL with cholesterol and lecithin as wall materials, forming a stable nanoscale delivery system. The nanoparticle exhibited uniform morphology, high encapsulation efficiency, satisfactory drug loading capacity, and sustained-release behavior, confirming its suitability as an effective carrier for CAR. CAR was identified as the most active compound among the tested essential oil formulations, and CAR@NL showed strong antifungal activity and favorable preservation effects against kiwifruit soft rot. DAPI staining indicated no obvious effect on fungal DNA, whereas PI staining confirmed that CAR@NL rapidly disrupted membrane integrity and caused severe hyphal damage, suggesting that membrane destruction is the primary antifungal mechanism. Moreover, flow cytometric analysis showed that CAR@NL induced only slight apoptosis in BEAS-2B cells at 50 mg/L compared with carbendazim, demonstrating its lower cytotoxicity toward mammalian cells. Therefore, CAR@NL represents a promising and safer alternative for the postharvest management of kiwifruit soft rot.

## Figures and Tables

**Table 1:** Fitting of different release models to the release behavior of CAR@NL.

Fitting Method	Equation	R^2^	Release Characterization Index (n)
Zero-Order Fitting	Y = 29.28 + 0.96t	0.86639	-
First-Order Fitting	Y = 80.05(1 − e^−0.047t^)	0.99855	-
Ritger–Peppas Fitting	Y = 9.95t^0.49^	0.96241	0.49
Higuchi Fitting	Y = 10.17t^1/2^ − 2.75	0.96442	-

**Table 2:** Regression equation for the virulence of Kiwifruit Soft Rot Pathogen.

Sample Name	Regression Equation	EC_50_ (mg/L)	R^2^	95% Confidence Limit (mg/L)
2% Acarenone water emulsion	Y = −2.84 + 1.31 × X	134.51	0.97	83.72~382.36
2% Cinnamaldehyde water emulsion	Y = −2.57 + 1.3 × X	92.66	0.978	63.92~196.38
2% CAR water emulsion	Y = −1.91 + 1.24 × X	34.90	0.961	28.71~45.99
2% CAR@NL	Y = −2.05 + 1.27 × X	41.76	0.973	33.87~57.95

## Data Availability

Data are available from the corresponding author upon reasonable request.

## References

[1] Rajan P., Natraj P., Kim M., Lee M., Jang Y.J., Lee Y.J., Kim S.C. (2024). Climate Change Impacts on and Response Strategies for Kiwifruit Production: A Comprehensive Review. Plants.

[2] Nazir M.F., Lou J., Wang Y., Zou S., Huang H. (2024). Kiwifruit in the Omics Age: Advances in Genomics, Breeding, and Beyond. Plants.

[3] Xiao T., Jia T., Wu W., Peng J., Pan L., Zhu X., Liu T., Cheng J., Wang H., Xiao L.J.H. (2025). Collection, Evaluation, and New Cultivar Breeding of Actinidia chinensis var. chinensis in Wudang Mountains, China. Horticulturae.

[4] Lawal H., Gaddafi M.S., Jamiu A.M., Edo G.S., Fremah O.G., El-Yakub A.U., Mahunu G.K., Wang K., Zhang H., Yang Q. (2025). Biocontrol and Nanotechnology Strategies for Postharvest Disease Management in Fruits and Vegetables: A Comprehensive Review. Foods.

[5] Liu G., Liu Q., Tan Q., Luo H., Xue W. (2024). Comparison of the effects of various preservation treatments on storage tolerance and soft rot resistance in kiwifruit: A network meta-analysis. Sci. Hortic..

[6] Song J., Shu Y., Zheng Y., Zhao Z., Long Y., Fan R. (2025). Construction and infection characteristics of a GFP-labelled Botryosphaeria dothidea causing kiwifruit soft rot disease. Plant Pathol..

[7] Li X., Zeng S., Liu J., Wang Y., Sui Y. (2022). Introduction and multiplex management strategies of postharvest fungal diseases of kiwifruit: A review. Biol. Control.

[8] Yue W., Xu J., Lv Z., Zhou C., Wu S., Zeng Y., Liu P. (2025). Biocontrol efficiency and the functional mechanisms of Bacillus velezensis RT-30 against kiwifruit soft rot. Hortic. Plant J..

[9] Wang H., Zhang X., Li K., Wu M., Zhuang Q., Li M., Garcia-Caparros P., Xie Y., Hu C., Liu M. (2025). Glycerol treatment enhances resistance to soft rot disease and maintains postharvest quality in kiwifruit. Postharvest Biol. Technol..

[10] Pan H., Zhong C., Xia L., Li W., Wang Z., Deng L., Li L., Long C. (2022). Antifungal activity of natamycin against kiwifruit soft rot caused by Botryosphaeria dothidea and potential mechanisms. Sci. Hortic..

[11] Musumeci T., Bonaccorso A., Carbone C. (2024). Basic concepts of liposomes: Components, structures, properties and classification. Liposomes in Drug Delivery.

[12] Chaurasiya A., Gorajiya A., Panchal K., Katke S., Singh A.K. (2022). A review on multivesicular liposomes for pharmaceutical applications: Preparation, characterization, and translational challenges. Drug Deliv. Transl. Res..

[13] Basak S., Das T.K. (2025). Liposome-based drug delivery systems: From laboratory research to industrial production—Instruments and challenges. ChemEngineering.

[14] Sengar A. (2025). Innovations and mechanisms in liposomal drug delivery: A comprehensive introduction. J. Adv. Nano Comput. Anal..

[15] Hamad I., Harb A.A., Bustanji Y. (2024). Liposome-Based Drug Delivery Systems in Cancer Research: An Analysis of Global Landscape Efforts and Achievements. Pharmaceutics.

[16] Ranjbar S., Emamjomeh A., Sharifi F., Zarepour A., Aghaabbasi K., Dehshahri A., Sepahvand A.M., Zarrabi A., Beyzaei H., Zahedi M.M. (2023). Lipid-based delivery systems for flavonoids and flavonolignans: Liposomes, nanoemulsions, and solid lipid nanoparticles. Pharmaceutics.

[17] Gandek T.B., van der Koog L., Nagelkerke A. (2023). A comparison of cellular uptake mechanisms, delivery efficacy, and intracellular fate between liposomes and extracellular vesicles. Adv. Healthc. Mater..

[18] Liu J., He Q., Lin X., Smagghe G. (2025). Recent progress in nanoparticle-mediated RNA interference in insects: Unveiling new frontiers in pest control. J. Insect Physiol..

[19] Bompard J., Rosso A., Brizuela L., Mebarek S., Blum L.J., Trunfio-Sfarghiu A.-M., Lollo G., Granjon T., Girard-Egrot A., Maniti O. (2020). Membrane fluidity as a new means to selectively target cancer cells with fusogenic lipid carriers. Langmuir.

[20] Rao Y., Tariq M., Wang M., Yu X., Liang H., Yuan Q. (2024). Preparation and characterization of bionics Oleosomes with high loading efficiency: The enhancement of hydrophobic space and the effect of cholesterol. Food Chem..

[21] Brandes A., Dunning M., Langland J. (2024). Antimicrobial activity of individual volatile compounds from various essential oils. Molecules.

[22] Li Y.-X., Erhunmwunsee F., Liu M., Yang K., Zheng W., Tian J. (2022). Antimicrobial mechanisms of spice essential oils and application in food industry. Food Chem..

[23] Liu Y., Qiu W., Mo Y., Tian J., Liao M., Jia B., Zhou Q., Liu F., Li X. (2025). Application of a Pickering Emulsion Stabilized by Zein and Cellulose Nanocrystalline Composite Particles to Preserve Kiwifruit. Molecules.

[24] Yu X., Wang J., Li X., Ma S., Zhu W., Wang H. (2023). Dual-responsive microcapsules with tailorable shells from oppositely charged biopolymers for precise pesticide release. Mater. Adv..

[25] Cen J., Li L., Huang L., Jiang G. (2022). Construction of a photothermal controlled-release microcapsule pesticide delivery system. RSC Adv..

[26] Cui S.F., Wang J.W., Li H.F., Fang R., Yu X., Lu Y.J. (2022). Microencapsulation of Capsaicin in Chitosan Microcapsules: Characterization, Release Behavior, and Pesticidal Properties against Tribolium castaneum (Herbst). Insects.

[27] Palvai S., Kpeglo D., Newham G., Peyman S.A., Evans S.D., Ong Z.Y. (2024). Free-standing hierarchically porous silica nanoparticle superstructures: Bridging the nano-to microscale for tailorable delivery of small and large therapeutics. ACS Appl. Mater. Interfaces.

[28] Lowe L.A., Kindt J.T., Cranfield C., Cornell B., Macmillan A., Wang A.J.S.M. (2022). Subtle changes in pH affect the packing and robustness of fatty acid bilayers. Soft Matter.

[29] Kurniawan J., Suga K., Kuhl T.L. (2017). Interaction forces and membrane charge tunability: Oleic acid containing membranes in different pH conditions. Biochim. Biophys. Acta (BBA)-Biomembr..

[30] Khan I., Bahuguna A., Kumar P., Bajpai V.K., Kang S.C. (2017). Antimicrobial Potential of Carvacrol against Uropathogenic Escherichia coli via Membrane Disruption, Depolarization, and Reactive Oxygen Species Generation. Front. Microbiol..

[31] Shen G., Qiu X., Hou X., Li M., Zhou M., Liu X., Chen A., Zhang Z. (2024). Development of Zanthoxylum bungeanum essential oil Pickering emulsions using potato protein-chitosan nanoparticles and its application in mandarin preservation. Int. J. Biol. Macromol..

[32] Pu Y., Chen L., Jiang W. (2024). Antimicrobial guar gum films optimized with Pickering emulsions of zein-gum arabic nanoparticle-stabilized composite essential oil for food preservation. Int. J. Biol. Macromol..

[33] Feng J., Chen Z., Chen W., Sun L., Yang J., He K., Dong S., Yuan S. (2022). Facile pathway to construct mesoporous silica nanoparticles loaded with pyraclostrobin: Physicochemical properties, antifungal activity, and biosafety. Pest. Manag. Sci..

[34] Liu G., Hou S., Tong P., Li J. (2022). Liposomes: Preparation, characteristics, and application strategies in analytical chemistry. Crit. Rev. Anal. Chem..

[35] Mehandole A., Walke N., Mahajan S., Aalhate M., Maji I., Gupta U., Mehra N.K., Singh P.K.J.A.P. (2023). Core–shell type lipidic and polymeric nanocapsules: The transformative multifaceted delivery systems. AAPS PharmSciTech.

[36] Andra V., Pammi S.V.N., Bhatraju L., Ruddaraju L.K. (2022). A Comprehensive Review on Novel Liposomal Methodologies, Commercial Formulations, Clinical Trials and Patents. Bionanoscience.

[37] Yousefi R., Rezaee Y., Bayat F., Rezaee E., Karami L., Dadashzadeh S., Haeri A. (2024). Molecular docking, molecular dynamics simulation, preparation, and characterization of naltrexone-phospholipid complex: A novel cargo with improved loading into multivesicular liposomes. Results Chem..

[38] Wang Y., Xie Y.H., Jiang Q.H., Chen H.T., Ma R.H., Wang Z.J., Yin M.Z., Shen J., Yan S. (2023). Efficient polymer-mediated delivery system for thiocyclam: Nanometerization remarkably improves the bioactivity toward green peach aphids. Insect Sci..

[39] Liu P., Chen G., Zhang J. (2022). A review of liposomes as a drug delivery system: Current status of approved products, regulatory environments, and future perspectives. Molecules.

[40] Eusepi P., Marinelli L., Borrego-Sánchez A., García-Villén F., Rayhane B.K., Cacciatore I., Viseras C., Di Stefano A. (2020). Nano-delivery systems based on carvacrol prodrugs and fibrous clays. J. Drug Deliv. Sci. Technol..

[41] Parys W., Dołowy M., Pyka-Pająk A. (2022). Significance of chromatographic techniques in pharmaceutical analysis. Processes.

[42] Pande S. (2023). Liposomes for drug delivery: Review of vesicular composition, factors affecting drug release and drug loading in liposomes. Artif. Cells Nanomed. Biotechnol..

[43] Walunj M., Doppalapudi S., Bulbake U., Khan W. (2020). Preparation, characterization, and in vivo evaluation of cyclosporine cationic liposomes for the treatment of psoriasis. J. Liposome Res..

[44] Haroosh H.J., Dong Y., Jasim S., Ramakrishna S. (2021). Improvement of drug release and compatibility between hydrophilic drugs and hydrophobic nanofibrous composites. Materials.

[45] Wang X., Swing C.J., Feng T., Xia S., Yu J., Zhang X. (2020). Effects of environmental pH and ionic strength on the physical stability of cinnamaldehyde-loaded liposomes. J. Dispers. Sci. Technol..

[46] Ren K., Cao X., Zheng L., Tian T., Zhang X., Dai J., Zhang H., Wang H., Jiang L. (2025). Pectin-modified 7S protein liposomes: Focus on structural properties, stability, and digestive properties loaded with Morin. Food Chem..

[47] Wang L., Huang X., Jing H., Ma C., Wang H. (2021). Bilosomes as effective delivery systems to improve the gastrointestinal stability and bioavailability of epigallocatechin gallate (EGCG). Food Res. Int..

[48] Li W., Long Y., Yin X., Wang W., Zhang R., Mo F., Zhang Z., Chen T., Chen J., Wang B. (2023). Antifungal activity and mechanism of tetramycin against Alternaria alternata, the soft rot causing fungi in kiwifruit. Pestic. Biochem. Physiol..

[49] Wu W., Yan C., Pan N., An J., Chen H., Fei Q., Xu S., Yang L.-L., Yang S., Chemistry F. (2025). Design and Synthesis of Ferulic Acid Derivatives with Trifluoromethyl Pyrimidine and Amide Frameworks for Combating Postharvest Kiwifruit Soft Rot Fungi. J. Agric. Food Chem..

